# 215 Targeted Stewardship and Infection Prevention Interventions reduced Clostridioides difficile rates at a Veterans Affairs Hospital

**DOI:** 10.1017/ash.2026.10598

**Published:** 2026-06-23

**Authors:** Bobby Warren, Guerbine Fils-aime, Aaron Barrett, Amanda Graves, Melissa Erickson, Deverick Anderson, Becky Smith, Matthew Steigel, Arielle Cohen

**Affiliations:** 1 Duke Center for Antimicrobial Stewardship and Infection Prevention; 2 Duke University Hospital; 3 Duke University Medical Center; 4 Duke University

## Abstract

**Background:** Deviations from expected relative humidity ranges in operating room (OR) sterile cores raise concern for compromise of sterile supply packaging and frequently result in large-scale disposal of supplies. Data guiding salvage versus discard decisions following humidity exposure are limited. Following two humidity events, we performed a two-experiment bench study to evaluate whether elevated ambient humidity or direct moisture exposure results in moisture penetration or microbial contamination of Tyvek-packaged sterile supplies. **Methods:** Experiment 1 evaluated direct moisture exposure by applying saturated compresses to package exteriors for 0.5, 1, 5, 30, or 60 minutes. Experiment 2 evaluated elevated ambient humidity by incubating packages at 37°C and 70% relative humidity without direct moisture contact. For Experiment 1, ambient temperature and relative humidity were continuously monitored, with humidity documented at swab timepoints (4, 8, 24, and 48 hours). For both experiments, moisture content of package exteriors and interiors was measured at baseline and serial timepoints up to 48 hours. Internal package surfaces were aseptically swabbed at each timepoint, plated onto general culture media, and incubated for 48 hours at 37°C. Negative controls without moisture or humidity exposure were included. **Results:** In Experiment 1, ten items were evaluated, including five exposed to direct compress application and five controls. External moisture content increased following compress exposure (range: 0.9–2.2) and decreased over time. Internal moisture content remained low across all samples, including controls (range: 0–1.4). No microbial growth was detected from any internal swab across all samples and timepoints (0/10 items; 0 CFU recovered). In Experiment 2, ten items were incubated under elevated ambient humidity. Occasional low-level external moisture readings were observed (maximum: 1.2), while internal moisture content remained 0 throughout the study period. All internal cultures were negative (0/10 items; 0 CFU recovered). **Conclusion:** Differences in absolute internal moisture measurements between experiments 1 and 2 reflect the use of a moisture meter reporting relative units influenced by ambient conditions and the fact that experiments were not conducted in parallel. Interpretations were therefore within experiments. Tyvek-packaged sterile supplies-maintained barrier integrity under both direct moisture exposure and sustained elevated ambient humidity, with no evidence of internal microbial contamination. These findings suggest that, in the absence of visible package compromise or internal condensation, salvage of Tyvek-packaged sterile supplies following humidity excursions may be reasonable. These findings provide bench-level evidence to support data-informed infection prevention decisions during OR humidity events and may help reduce unnecessary waste and operational disruption.

